# Safeness of Diets Based on Gluten-Free Buckwheat Bread Enriched with Seeds and Nuts—Effect on Oxidative and Biochemical Parameters in Rat Serum

**DOI:** 10.3390/nu12010041

**Published:** 2019-12-22

**Authors:** Michal Świeca, Julita Regula, Joanna Suliburska, Urszula Zlotek, Urszula Gawlik-Dziki, Isabel M. P. L. V. O. Ferreira

**Affiliations:** 1 Department of Biochemistry and Food Chemistry, University of Life Sciences, Skromna Str. 8, 20-704 Lublin, Poland; michal.swieca@up.lublin.pl (M.Ś.); urszula.zlotek@up.lublin.pl (U.Z.); urszula.gawlik@up.lublin.pl (U.G.-D.); 2 Institute of Human Nutrition and Dietetics, Poznan University of Life Sciences, Wojska Polskiego Str. 31, 60-624 Poznan, Poland; joanna.suliburska@up.poznan.pl; 3 LAQV/REQUIMTE, Laboratório de Bromatologia e Hidrologia, Departamento de Ciências Quıímicas, Faculdade de Farmácia, Universidade do Porto, 4051-401 Porto, Portugal; isabel.ferreira@ff.up.pt

**Keywords:** antioxidant status, bread enrichment, buckwheat, gluten free bread, lipid profile

## Abstract

Buckwheat breads enriched with seeds (e.g., poppy, carum, amaranth, sunflower, and pumpkin) and nuts can be excellent sources of selected macro- and microelements and bioactive components, such as phenolics, essential oils, unsaturated fatty acids, fiber, and vitamins; however, no studies described their impacts on body biochemical parameters and antioxidant status. The aim of this study was to a determine the safety (the analyses of blood morphological and biochemical parameters) of short-term diets based on buckwheat breads supplemented with the commonly used functional ingredients. Additionally, we confirmed the usefulness of these fortified breads in a reduction of blood cholesterol and triacylglycerols, as well as an improvement of in vivo antioxidant status of Wistar rats. Enriched breads presented an increased phenolic content; however, it has not been translated into an elevation of antioxidant capacities. During short-term in vivo experiments, the studied breads increased the body mass of the rats, except the control buckwheat bread. Compared to the control, the poppy-milk bread markedly lowered (–23%) and egg yolk–carum bread significantly increased (+17%) the total cholesterol concentration in serum. All the fortified breads decreased triacylglycerols’ levels by about 50%. Bread enriched with the poppy–milk, milk-seed, egg yolk–carum, and a mix of additives decreased superoxide dismutase activity by 68%, 66%, 73%, and 71%, respectively. Catalase activity was significantly decreased in the rats fed with carum bread (–62%) and markedly increased in the groups fed with egg yolk–carum bread (+89%), hazel nuts–amaranth bread (+72%), and milk–seeds bread (+65%). The results confirmed the usefulness and safety of functional additives in buckwheat breads.

## 1. Introduction

Bread is the most popular staple food in the world due to its nutritive value, low price, and simplicity of usage and production. Bread is mainly produced from wheat and rye flours; however, in the last years, there has been a growing interest in alternative crops (amaranth, quinoa, buckwheat, etc.) [1]. This trend results from two main reasons. In populations, there is a group of people for whom consumption of prolamins, protein fraction commonly present in wheat, rye, barley, and oats, causes some disorders e.g., coeliac disease, allergy, and non-celiac sensitivity to gluten [2]. On the other hand, in modern communities, there is a kind of trend toward adopting a gluten-free diet, which is pointed out as promoting well-being and improving consumers’ health [1,3].

The main alternative gluten-free crops for bread production are amaranth, quinoa, rice, and buckwheat [1]. Buckwheat, belonging to the *Polygonaceae* family, has received increasing attention as a potential functional food not only for bread, but also for cookies, pasta, noodles, and beer [1]. It is a rich source of starch (55–70%), including resistant starch (30–35%) [3,4] and proteins (8–19%) of high biological quality [3]. Additionally, it also contains valuable trace elements (zinc, copper, manganese, iron, potassium, phosphorus, magnesium, boron, cobalt, and platinum) and dietary fiber [3,5]. Buckwheat is also a rich source of phenolics, such as rutin, quercetin, quercitrin, orientin, homoorientin, vitexin, and isovitexin—flavonoids with well-documented pro-health properties [6,7].

So far, buckwheat flour has been successfully added to many food products, to increase flavonoid, mineral, and resistant starch contents [8]. To meet consumers’ needs and preferences, some optional ingredients can be added to enhance nutritional and nutraceutical quality and also improve organoleptic characteristics. Buckwheat breads enriched with seeds (e.g., poppy, carum, amaranth, sunflower, and pumpkin) and nuts, can be excellent sources of selected macro- and microelements and bioactive components, such as phenolics, essential oils, unsaturated fatty acids, fiber, and vitamins [8]; however, so far, there are no studies that describe their safeness and impact on body biochemical parameters and antioxidant status. A wide range of studies concerning the safety of diets enriched with functional ingredients and evaluation of potential positive and/or negative effects of such products is of major relevance. These studies were designed to investigate the safety (the analyses of blood morphological and biochemical parameters) of short-term diets containing buckwheat breads supplemented with the commonly used functional ingredients. Additionally, they are aimed to confirm the usefulness of these fortified breads in a reduction of blood cholesterol and triacylglycerols, as well as in an improvement of in vivo antioxidant status of Wistar rats. 

## 2. Materials and Methods

### 2.1. Bread Preparation

All breads analyzed in this study were produced from buckwheat. Natural components commonly available on the market were used to enrich the buckwheat bread: B1—bread enriched with milk and poppy; B2—bread enriched with carum, amaranth, and seeds; B3—bread enriched with hazel nuts, seeds, and amaranth flour; B4—bread enriched with milk, amaranth flour, and seeds; B5—bread enriched with amaranth flour, seeds, yolk, and carum; B6—bread enriched with milk, amaranth flour, seeds, and poppy. Buckwheat bread was used as the control bread (BC), without supplements (Table 1). To prepare the bread, the following ingredients were also used: potato and corn starch (Glutenex Company, Sady, Poland), rapeseed oil (Kruszwica, Poland), salt (Solino, Poland), fresh yeast (*Saccharomyces cerevisiae*) (Lesaffre, Warsaw, Poland), and saccharose (Pfeifer & Langen, Gostyn, Poland), milk powder (SM Mlekovita, Wysokie Mazowieckie, Poland), poppy seeds (VOG, Skierniewice, Poland), sunflower seeds (VOG, Skierniewice, Poland), flax seeds (Bio Planet, Leszno (Masovian Voivodeship), Poland), pumpkin seeds (VOG, Skierniewice, Poland), egg yolk (Ovovita, Rzgow. Poland), carum (KOTÁNYI, Warsaw, Poland), hazel nuts (VOG, Skierniewice, Poland), and amaranth (Bio Planet, Leszno (Masovian Voivodeship), Poland).

### 2.2. Animals and Diets

The study was conducted for 14 days on 42 male rats (6 weeks old) of the albino Wistar strain, with a mean body weight of 234.1 g ± 25.6. The experiment was performed with the agreement of the local bioethics committee (approval no. 888/11). Animals were kept in a thermostatically controlled room (22 °C ± 2), in a 12 h light/dark cycle, at 55–60% humidity, with unlimited access to distilled water (ad libitum). The animals were divided into seven groups. They were fed an AIN93M diet [9] with a 70% addition of gluten-free breads (control group BC—diet enriched with bread without functional supplements) and six group with breads enriched with supplements (B1—bread enriched with milk and poppy; B2—bread enriched with carum, amaranth, and seeds; B3—bread enriched with hazel nuts, seeds, and amaranth flour; B4—bread enriched with milk, amaranth flour, and seeds; B5—bread enriched with amaranth flour, seeds, yolk, and carum; B6—bread enriched with milk, amaranth flour, seeds, and poppy). The food consumption was recorded daily, and the animals were weighed once a week. At the completion of the feeding period on the last day of the experiment, after 12 h of fasting, the animals were euthanized with a sodium thiopental injection (40 mg/kg body weight). Blood was then collected from the left heart chamber.

Energy and nutrients intake were calculated according to USDA National Nutrient Database [10]. The nutritional value of the diets with gluten-free breads is shown in Table 2.

### 2.3. Gastrointestinal Digestion In Vitro

Evaluation of total phenolic content and antioxidant capacity of breads was performed on the bio-accessible fraction obtained after in vitro simulation of gastrointestinal digestion [4].

### 2.4. Analysis of Blood Morphological and Biochemical Parameters

The blood was collected by cardiac puncture in tubes with heparin sodium, to obtain whole blood for morphological tests and in serum-separated tubes for biochemical parameters. The coagulated blood was left to clot at room temperature for 30 min, and then it was centrifuged for 15 min at 3600× *g*. The following morphological and biochemical parameters were determined: white blood cells (WBC), mean corpuscular volume (MCV), mean corpuscular hemoglobin (MCH), mean corpuscular hemoglobin concentration (MCHC), lymphocytes (LYM), platelet distribution width (PDW), red blood cell distribution width (RDW), mean platelet volume (MPV), glucose (GLU), albumins (ALB), triacylglycerols (TAG), alanine transaminase activity (ALA), aspartate transaminase activity (AST), total cholesterol (TCH), and high-density lipoprotein cholesterol (HDL). Values for the morphological indices were determined by using a Sysmex K-1000 hematological analyzer (TAO Medical Electronics Co., Kobe, Japan) according to the standard procedures. The concentration of glucose in the blood serum was estimated by the glucose oxidase method. Total cholesterol and triglyceride levels in serum were measured by using commercial kits (Randox Laboratory Ltd., UK). Albumin was measured by the immunoassay method, using a rat kit. The activities of liver enzymes, such as ALT and AST, were determined according to Dembinska-Kiec and Nastalski [11].

### 2.5. Total Phenolics Content

Total phenolics content in bread was estimated according to the Folin–Ciocalteu method [12]. The amount of total phenolics was expressed as gallic acid equivalents (GAE).

### 2.6. Antioxidant Capacity

#### 2.6.1. Antiradical Activity (ABTS)

The ability to quench free radicals of digested bread extracts and rat serum were determined by using an improved ABTS decolorization assay [13]. Antiradical activity was expressed as a Trolox equivalent. 

#### 2.6.2. Ferric Reducing Power (FRAP) 

Reducing powers of digested bread extracts and rat serum were determined by the method of Oyaizu [14]. Reducing power was expressed as a Trolox equivalent.

#### 2.6.3. Catalase Activity Assay (CAT)

CAT activity was assayed by the method of Claiborne [15] with some modification [16]. The catalase activity was expressed in U, where 1U was defined as the amount of enzyme present that decomposed 1 μmol of H_2_O_2_ per min (method conditions). 

#### 2.6.4. Superoxide Dismutase Assay (SOD)

SOD activity was determined by using a kinetic mode [17]. The SOD activity was expressed in U, where 1U was defined as the amount of enzyme present that produced a change in absorbance of 0.001 per min (method conditions).

### 2.7. Statistical Analysis

Statistical tests were performed by using Statistica 6.0 software (StatSoft, Inc., Tulsa, OK, USA). All data were tested for normal distribution by use of the Shapiro–Wilk test. Normally distributed values were compared, using ANOVA. Means of analyzed traits in the groups were compared, using one-way analysis of variance, while intergroup differences were assessed by Tukey’s post hoc test, at the significance level *p* < 0.05. Data were evaluated by using Pearson’s correlation coefficients, to identify relationships between selected features**.**

## 3. Results and Discussion

Epidemiological studies indicate an inverse relationship between a high-fiber diet, as well as dietary flavonoids intake and the risk of many disorders, including cardiovascular disease, cancers, and inflammations [8,18]. Many studies concerning the composition of buckwheat confirm that these gluten-free seeds present high contents of polyphenols and resistant starch, being an interesting ingredient to be used in functional diets. 

**Figure 1:**
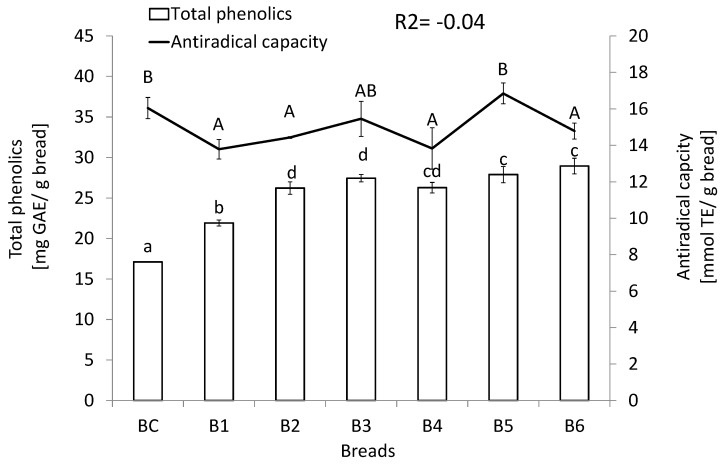
Phenolics content and antiradical capacity of the potential bio-accessible fraction of studied breads. Means (*n* = 6, ± standard deviation) followed by different lower-case letters are significantly different at *p* < 0.05. BC—control bread; B1—bread enriched with milk and poppy; B2—bread enriched with carum, amaranth, and seeds; B3—bread enriched with hazel nuts, seeds, and amaranth flour; B4—bread enriched with milk, amaranth flour, and seeds; B5—bread enriched with amaranth flour, seeds, yolk, and carum; B6—bread enriched with milk, amaranth flour, seeds, and poppy; TE—Trolox equivalent; GAE—gallic acid equivalent.

The level of potentially bio-accessible phenolics, as well as antiradical activity quantified after in vitro simulation of gastrointestinal digestion of the studied breads, is depicted in Figure 1. Breads enriched with seeds and grains presented an increased phenolic content; however, it has not been translated into an elevation of their antioxidant capacities, since there was no correlation between both features. 

Buckwheat-enriched wheat breads are characterized by the increased antioxidant potential due to the presence of rutin [8]. Compared to the previous studies by Świeca et al., [16] concerning rice-based gluten-free bread enriched with functional supplements (analogical to those used in this study) higher phenolic contents and antioxidant capacity was observed in the extracts obtained after in vitro simulation of gastrointestinal digestion of buckwheat breads. However, concerning buckwheat breads the improvement of antioxidant potential was not correlated with the increase of phenolics. It may be due to fact that rice flour contains about 40 times less of polyphenolic than that obtained from buckwheat [19], and the added value (antioxidant capacity of supplements) was masked by extremely high antioxidant potential of buckwheat flour. Additionally, the baking process leads to the formation of new heat-generated compounds, mainly by *Maillard* reactions, and bread is a complex system. Thus, the use of different ingredients had a variable impact on the overall antioxidant capacity [19]. The basic buckwheat bread (BC) and the studied supplemented bread were characterized by significantly higher antioxidant potential when compared to the wheat bread [8].

In this study, diets enriched with functional components caused an increase of body mass—Table 2 (basic buckwheat breads, which are low in fat and calories), which may be explained by the fact that nuts and seeds are typically high in dietary fat; thus, fortified breads presented increased caloric value, as confirmed by the theoretical calculation performed, using standard values taken from the USDA National Nutrient Database (Table 1). However, according to the last studies of Nishi et al. [18], these ingredients are considered to be heart-healthy foods. Additionally, this assumption is supported by the results concerning the lipids profile observed after experiment (Table 3).

The studied gluten-free breads did not significantly influence most of the morphological parameters of rat blood; however, a significant increase in concentration of thrombocytes was observed in the group with poppy–milk bread (Table 4). The relevant changes were observed in the lipid profiles of serum (Table 3). In comparison with BC, the animals fed with B1, B3, and B6 presented a decrease in total cholesterol concentration; however, these changes were significant only in the case of the B1 diet. An adverse effect was observed for the B5 diet. Most importantly, rats fed with all the enriched breads had significantly lower levels of triacylglycerols (compared to the BC diet). The lowest levels were determined for B3, B4, and B6 (reduction of ~50%–55% in regard to BC) (Table 4). 

The antioxidant status of rats fed with enriched breads was determined based on the reducing and antiradical abilities of serum, and changes in the activity of two enzymes involved in enzymatic antioxidant defense (Table 5). No statistically significant changes, affected by the studied diets, were observed in reducing and antiradical potential of rat serum. Serum superoxide dismutase activity was significantly reduced by all the studied diets (except B2 and B3). Serum catalase activity was significantly increased in rats fed with B3, B4, and B5 by about 90%, 70%, and 65%, respectively, in comparison with BC. Contrary to these results, the B2 diet promoted a significant reduction in the catalase activity. The observed changes cannot be directly explained by a higher amount of phenolics introduced to diet by functional additives. It may be speculated that other factors, such as microbiota action (e.g., release of bound phenolics and restructuration of phenolics structure), interaction of phenolics with food components (protein- and starch-phenolics interactions), and release of bioactive peptides from proteins, play an important role in the creation of final effects. 

The beneficial effects observed after consumption of buckwheat or buckwheat-enriched food products is well documented in animal and human studies [20]. Stokić et al., [21] reported that buckwheat protein extract reduces hepatic triacylglycerols concentration, adipose tissue weight, and hepatic lipogenesis in rats. The diet containing buckwheat-enriched wheat bread (at the level of 50%) was also tested in normal-weight patients on statin therapy over one-month dietetic intervention. In comparison with the wheat bread, the enriched breads were characterized by 2.22 times higher total dietary fiber and 4.29 times higher total phenolics content and significantly decreased total and LDL-cholesterol. According to the literature data, buckwheat can reduce the concentration of cholesterol in the serum by increasing the fecal excretion of steroids, which is induced by the binding of steroids to undigested proteins [1]. Additionally, Świeca [4] reported that a high amount of flavonoids in buckwheat flour significantly decreases the digestibility of starch and proteins by formation of undigested complexes with both digestive enzymes and buckwheat proteins. The presence of such interactions in the phenolics-rich food has already been reported [22]. On the other hand, lowering of cholesterol content by buckwheat-rich diet may be due to a high content of dietary fiber, which effectively binds bile acid (produced from cholesterol) [23]. In comparison with previous studies performed with gluten-free rice-based breads and using similar ingredients [16], the lipid profile was not significantly different, although significant differences were found in the activities of AST and ALT. It is known that a buckwheat-rich diet may promote an increase in the activity of these liver markers that is linked with intensification of cholesterol metabolism [24]; however, in this study, such effect was not observed (compared to wheat or other gluten-free bread) [16]. Most importantly, similarly to the previous studies by Lee et al. [7], in the rats fed with the enriched bread, a decrease of ALT activity was observed (statistically insignificant, but a tendency was clearly visible).

An increase of the antioxidant status of rats after consumption of standard diets including bread crust glycated compounds with a high antioxidant potential was also previously described by Pastoriza et al. [25]. A positive effect of a diet enriched with buckwheat was also confirmed by Zduńczyk et al. [26]. Compared with the diet containing naked oat, a higher antioxidant capacity in vivo of the diet with buckwheat waste increased numerically, but not statistically, the activity of glutathione peroxidase and the level of TBARS. A similar observation was also found during the study concerning cardioprotective effects of diet with different grains on lipid profiles and antioxidative potential in obesity-induced rats [27]. Compared to the other studied diets, the buckwheat-rich diet caused a significant reduction of plasma TBARS and low-density lipoprotein cholesterol (LDL-C) (*p* < 0.05) and increased high-density lipoprotein (HDL-C). In summary, the basic buckwheat bread (BC) and the studied supplemented bread were characterized by a significantly higher antioxidant potential [8].

## 4. Conclusions

The results in this short-term experiment confirmed the usefulness and safety of functional ingredients for the production of bakery goods based on buckwheat flour. Breads enriched with functional ingredients have a beneficial influence on triacylglycerols concentration in the serum of rats, although the physiological relevance of our observations should be further examined in long-term studies on experimental animals and/or humans.

## Figures and Tables

**Table 1:** Buckwheat breads’ composition.

Ingredients (%)	Breads						
	BC	B1	B2	B3	B4	B5	B6
buckwheat flour	20.7	19.7	17.2	17.7	17.2	17.4	16.6
corn and potato starch (% potato)	31.1 (20.7)	24.2 (14.3)	19.4 (10.8)	19.9 (11.1)	19.4 (10.8)	19.6 (10.9)	18.7 (10.4)
*Saccharomyces cerevisiae*	2.6	2.5	2.2	2.2	2.2	2.2	2.1
saccharose	3.1	3.0	2.6	2.7	2.6	2.6	2.5
NaCl	0.5	0.5	0.4	0.4	0.4	0.4	0.4
rapeseed oil	0.5	0.5	0.4	0.4	0.4	0.4	0.4
milk powder	-	3.0	-	-	2.6	-	2.5
amaranth flour	-	-	2.6	2.7	2.6	2.6	2.5
flax	-	-	2.2	2.2	2.2	2.2	2.1
sunflower seeds	-	-	2.2	2.2	1.7	2.2	2.1
pumpkin seeds	-	-	2.2	2.2	2.2	2.2	2.1
hazel nuts	-	-	1.7	2.7	1.7	2.2	2.1
poppy	-	7.4	-	2.2	2.6	-	4.1
egg yolk	-	-	0.4	-	-	0.9	0.4
carum	-	-	4.3	-	-	2.6	0.0
water	41.5	39.4	42.2	42.5	42.2	42.6	41.5

BC—control bread; B1—bread enriched with milk and poppy; B2—bread enriched with carum, amaranth and seeds; B3—bread enriched with hazel nuts, seeds, and amaranth flour; B4—bread enriched with milk, amaranth flour, and seeds; B5—bread enriched with amaranth flour, seeds, yolk, and carum; B6—bread enriched with milk, amaranth flour, seeds, and poppy.

**Table 2:** Approximate energy, fiber, fat, and unsaturated fatty acids intake and mean body mass in rats.

	Energy *(kcal)	Fiber * (%)	Fat * (%)	Unsaturated Fatty Acids * (%)	Body Mass Change (g)
BC	166.90	3.29	1.71	0.23	–14.67 ± 2.58 a
B1	191.16	4.47	3.40	2.33	3.67 ± 10.67 b
B2	190.32	4.55	6.78	0.99	4.33 ± 5.16 b
B3	197.00	4.26	6.82	1.42	5.67 ± 6.02 b
B4	196.00	4.29	6.44	1.45	10.67 ± 8.02 b
B5	187.31	4.22	6.58	0.95	9.50 ± 4.51 b
B6	204.72	4.53	7.31	1.97	7.75 ± 7.58 b

BC—control bread; B1—bread enriched with milk and poppy; B2—bread enriched with carum, amaranth, and seeds; B3—bread enriched with hazel nuts, seeds, and amaranth flour; B4—bread enriched with milk, amaranth flour, and seeds; B5—bread enriched with amaranth flour, seeds, yolk, and carum; B6—bread enriched with milk, amaranth flour, seeds, and poppy. Means (*n* = 6, ± standard deviation) followed by different lower-case letters between breads are significantly different at *p <* 0.05. * Calculated according to USDA National Nutrient Database.

**Table 3:** Influence of buckwheat bread on biochemical parameters in rats.

		Diet					
	BC	B1	B2	B3	B4	B5	B6
GLU (mg dL**^−^**^1^)	117.8 ± 21.7	139.5 ± 33.5	118.8 ± 26.1	111.0 ± 18.8	102.2 ± 23.5	130.8 ± 26.9	109.7 ± 12.3
ALB (g dL**^−^**^1^)	3.37 ± 0.1	3.58 ± 0.1	3.60 ± 0.1	3.67 ± 0.1	3.63 ± 0.2	3.60 ± 0.2	3.53 ± 0.1
TCH (mg dL**^−^**^1^)	83.5 ± 7.2 b	69.3 ± 3.1 a	90.1 ± 14.3 bc	76.8 ± 9.5 ab	90.0 ± 11.8 bc	97.6 ± 5.8 c	79.5 ± 11.8 ab
HDL (mg dL**^−^**^1^)	29.4 ± 3.6 ab	24.8 ± 1.8 a	31.0 ± 4.1 ab	26.1 ± 2.7 ab	29.9 ± 3.4 ab	32.5 ± 3.2 b	27.8±1.5 ab
TAG (mg dL**^−^**^1^)	42.5 ± 5.9 c	27.8 ± 4.8 b	22.0 ± 5.5 ab	20.0 ± 4.5 ab	18.3 ± 3.7 a	28.1 ± 12.1 ab	18.1 ± 5.8 ab
AST (U L**^−^**^1^)	93.3 ± 34.7	98.0 ± 41.1	74.5 ± 22.0	90.6 ± 39.3	83.5 ± 32.2	91.5 ± 45.4	86.3 ± 42.2
ALT (U L**^−^**^1^)	27.6 ± 8.9	23.1 ± 7.4	21.1 ± 8.8	19.5 ± 8.3	16.1 ± 3.7	20.0 ± 6.3	21.8 ± 6.8

Means (*n* = 6, ± standard deviation) followed by different lower-case letters within rows are significantly different at *p* < 0.05. BC—control bread; B1—bread enriched with milk and poppy; B2—bread enriched with carum, amaranth, and seeds; B3—bread enriched with hazel nuts, seeds, and amaranth flour; B4—bread enriched with milk, amaranth flour, and seeds; B5—bread enriched with amaranth flour, seeds, yolk, and carum; B6—bread enriched with milk, amaranth flour, seeds, and poppy; GLU—glucose; ALB—albumins; TCH—total cholesterol TAG—triacylglycerols; ALT—alanine transaminase; AST—aspartate transaminase; HDL—high-density lipoprotein cholesterol.

**Table 4:** Effects of bread diets on the blood morphological parameters.

	Breads						
	BC	B1	B2	B3	B4	B5	B6
WBC (×10^3^ µL**^−^**^1^)	2.45 ± 0.87	3.62 ± 0.76	3.92 ± 1.28	3.26 ± 0.63	3.32 ± 0.83	2.72 ± 0.99	3.35 ± 0.80
Erythrocytes (×10^6^ µL**^−^**^1^)	7.19 ± 0.37	7.34 ± 0.20	7.16 ± 0.23	7.33 ± 0.24	7.35 ± 0.20	7.41 ± 0.24	7.19 ± 0.40
Hemoglobin (g dL**^−^**^1^)	14.15 ± 0.68	14.07 ± 0.53	14.07 ± 0.40	14.08 ± 0.42	14.32 ± 0.64	14.18 ± 0.33	13.92 ± 0.62
Hematocrit (%)	41.87 ± 2.81	42.17 ± 1.42	41.62 ± 1.60	41.80 ± 0.83	42.52 ± 1.96	42.67 ± 1.24	41.50 ± 2.46
MCV (fl)	58.28 ± 2.72	57.47 ± 1.18	58.08 ± 1.25	57.04 ± 1.99	57.82 ± 2.16	57.60 ± 1.31	57.70 ± 1.38
MCH (pg)	19.70 ± 0.83	19.17 ± 0.51	19.63 ± 0.36	19.22 ± 0.80	19.47 ± 0.72	19.15 ± 0.19	19.35 ± 0.56
MCHC (g dL**^−^**^1^)	33.83 ± 0.98	33.37 ± 0.45	33.85 ± 1.14	33.68 ± 0.37	33.68 ± 0.29	33.25 ± 0.63	33.57 ± 0.69
Thrombocytes (×10^3^ µL**^−^**^1^)	773 ± 62.1 a	923 ± 71.5 b	838 ± 54.5 ab	859 ± 94.1 ab	876 ± 64.4 ab	826 ± 105.4 ab	799 ± 98.0 ab
LYM (%)	93.88 ± 1.02	94.58 ± 0.95	89.35 ± 8.28	93.50 ± 1.17	94.73 ± 1.14	93.35 ± 1.53	93.50 ± 1.37
LYM (×10^3^ µL**^−^**^1^)	2.42 ± 0.93	3.43 ± 0.70	3.53 ± 1.28	3.02 ± 0.58	3.15 ± 0.80	2.53 ± 0.90	3.13 ± 0.77

Means (*n* = 6 ± standard deviation) followed by different lower-case letters within row are significantly different at *p* < 0.05. BC—control bread; B1—bread enriched with milk and poppy; B2—bread enriched with carum, amaranth, and seeds; B3—bread enriched with hazel nuts, seeds, and amaranth flour; B4—bread enriched with milk, amaranth flour, and seeds; B5—bread enriched with amaranth flour, seeds, yolk, and carum; B6—bread enriched with milk, amaranth flour, seeds, and poppy; WBC—white blood cells; MCV—mean corpuscular volume; MCH—mean corpuscular hemoglobin; MCHC—mean corpuscular hemoglobin concentration; LYM—lymphocytes.

**Table 5:** The antioxidant status of rats’ serum after feeding with buckwheat breads.

	Catalase Activity (kU dL^−1^)	SOD Activity (kU dL^−1^)	Antiradical Activity (mol TE dL^−1^)	Reducing Potential (molTE dL^−1^)
BC	67.67 ± 20.9 b	16.24 ± 2.4 e	273.8 ± 37.1 ab	36.1 ± 0.83
B1	61.03 ± 7.7 b	5.27 ± 1.6 bc	299.5 ± 8.3 b	36.3 ± 4.49
B2	25.92 ± 4.6 a	16.11 ± 0.7 e	298.1 ± 15.6 ab	36.9 ± 0.73
B3	116.09 ± 13.3 cd	10.63 ± 1.9 de	306.0 ± 12.1 b	35.8 ± 3.29
B4	111.49 ± 17.2 cd	5.44 ± 0.9 bc	307.8 ± 10.2 ab	37.6 ± 3.70
B5	128.16 ± 20.1 d	4.43 ± 1.1 abc	298.4 ± 19.2 ab	37.7 ± 0.42
B6	80.46 ± 18.9 bc	4.71 ± 1.2 abc	287.6 ± 4.2 a	39.1 ± 2.66

Means (*n* = 6, ± standard deviation) followed by different lower-case letters in columns are significantly different at *p* < 0.05. BC—control bread; B1—bread enriched with milk and poppy; B2—bread enriched with carum, amaranth and seeds; B3—bread enriched with hazel nuts, seeds, and amaranth flour; B4—bread enriched with milk, amaranth flour, and seeds; B5—bread enriched with amaranth flour, seeds, yolk, and carum; B6—bread enriched with milk, amaranth flour, seeds, and poppy; TE—Trolox equivalent.
